# Integrating NSGA-II and TOPSIS for Stacking Model Optimization in Pursuit of Halide Double Perovskite Screening

**DOI:** 10.3390/ma19102018

**Published:** 2026-05-12

**Authors:** Guiqin Liang, Jian Zhang

**Affiliations:** 1 College of Information and Communication, Guilin University of Electronic Technology, Guilin 541004, China; 2 College of Physics and Electronic Information Engineering, Guilin University of Technology, Guilin 541004, China; 3 College of Materials Science and Engineering, Guilin University of Electronic Technology, Guilin 541004, China; 4 Guangxi Key Laboratory of Information Materials, Guangxi Collaborative Innovation Center of Structure and Property for New Energy and Materials, Guilin 541004, China

**Keywords:** stacking model optimization, TOPSIS, NSGA-II, SHAP, halide double perovskite

## Abstract

Halide double perovskite materials have been used for various applications; their bandgap (E_g_) and heat of formation (ΔH_f_) are their key properties. They can be obtained through calculations based on high-throughput density functional theory (DFT), but such calculations are computationally expensive and time-consuming. Machine learning (ML) has proved to be an effective tool for screening potential materials. The prediction accuracy of ML models strongly depends on both input features and ML algorithms. However, there is no unified feature set with which ML models can effectively distinguish halide double perovskite materials. Although it has been proven that stacking ML models can achieve higher prediction accuracy than individual ML models, little attention has been paid to the optimization of stacking models. To solve these problems, we constructed a new feature set obtained from periodic tables for predicting the E_g_ and ΔH_f_ of halide double perovskites, and we further proposed a method integrating the nondominated sorting genetic algorithm (NSGA-II) and the Technique for Order Preference by Similarity to Ideal Solution (TOPSIS) decision-making tool for stacking model optimization to predict the E_g_ and ΔH_f_ of 540 compounds of halide double perovskites. Experimental results from 40 runs of 5-fold cross-validation demonstrate that our proposed new feature set enables ML models to achieve better performance than the original feature set. Moreover, the stacking model optimized by our proposed method yields better predicting performance than that of any individual single model and stacking regression models without optimization, with average improvements of 5.02%, 2.70%, 3.72% and 0.28% in MSE, RMSE, MAE and R^2^, respectively, in E_g_ prediction, thus providing more effective guidance for screening potential compounds for solar cells from a large quantity of materials.

## 1. Introduction

Recently, machine learning (ML) has enabled significant progress in material design and discovery, including hybrid organic–inorganic perovskites [1,2] and inorganic double perovskites [3,4]. Halide double perovskite materials have been applied to various fields, including solar cells, light-emitting diodes, catalysts, batteries, and photodetectors, which are mainly categorized by their bandgap (E_g_) [5,6]. Recent experimental efforts have further advanced perovskite photovoltaic performance through optimized fabrication and device architecture [7,8]. For instance, Barar et al. employed a two-diode model combined with the Lambert W function to extract the key performance parameters of perovskite solar cells [9]. In addition, the stability of compounds can also be taken into consideration and is indicated by the heat of formation (ΔH_f_) [10].

Traditionally, E_g_ and ΔH_f_ are usually obtained from calculations based on DFT in a traditional way, which is computationally expensive and time-consuming [11,12], making it impossible to apply to a large database. To solve this problem, an ML technique driven by data has been employed to extract potential materials from databases, accelerating the process of material design [5,13,14,15]. The identification of an accurate input feature set strongly correlates with the performance of ML models, and different feature sets have an influence on the accuracy of ML models to some extent [16,17,18]. Furthermore, the feature sets mentioned in previous works were constructed randomly by researchers.

Agiorgousis et al. [16] selected only three features from the periodic table—ionization potential (IP), Pauling electronegativity (EN), and atomic radius (AR)—as input features for bandgap prediction. However, the average bandgap errors were as high as 0.457 eV for the training set and 0.514 eV for the testing set based on Random Forest regression. Such prediction accuracy is insufficient for reliably identifying suitable materials based on bandgap values. Although Im et al. [10] predicted the E_g_ and ΔH_f_ of halide double perovskite with 32 input features, achieving an average RMSE as low as 0.221 eV for E_g_ and 0.023 eV/atom for ΔH_f_, input features (e.g., distance between cations at A-, $B^{+}$-, and $B^{3+}$-site, and anions at the X-site) with top feature importance scores need to be obtained from DFT modeling, which requires significant computational cost and enormous computation time [19], making it difficult to apply to screening a large amount of new materials. The desire to easily apply ML models for property prediction based on a new database with high performance accuracy calls for a more proper and convenient input feature set.

Additionally, the selection of an appropriate ML algorithm also has a significant impact on predictive performance. Most previous studies [10,16,20,21,22,23,24] utilized single regression models to predict the properties of perovskite materials, and the prediction accuracy needs to be further improved. Ensemble learning is a common approach for improving overall prediction performance, and it can be implemented by integrating multiple base learners in a certain way to achieve higher prediction accuracy and effectively reduce overfitting risks associated with individual models [25]. Chen et al. [26] proposed an integrated model called R-X-S by combining ridge regression (RR), eXtreme Gradient Boosting (XGBR), and support vector regression (SVR) to predict the melting point of low-melting-point alloys with a low root mean squared error (RMSE) and high correlation coefficient (R) calculated from the simple average results of three models, and it achieved better performance than individual models. Lu et al. [27] developed a weighted voting regressor model to predict the bandgap of hybrid organic–inorganic perovskites (HOIPs) with lower RMSE, which included four sub-models: CATBoost, XGBoost, LightGBM and Gradient Boosting (GBT).

Moreover, ensemble ML models with stacking methods can achieve better prediction results than other ensemble methods (viz. voting, boosting and bagging) and have been widely used in various applications due to their excellent performance [28,29,30]. It has been found that stacking ensemble learning algorithms can achieve better prediction performance than individual ML models. Meharie et al. [31] proposed a stacking ensemble model with a combination of three models, including linear regression (LR), SVR, and artificial neural networks (ANNs) as base models and GBT as a meta-regressor for predicting the final project cost. Comparison results revealed that the stacking ensemble model outperforms single models. Asante-Okyere et al. [32] developed a stacking model composed of multivariate adaptive regression spline (MARS), GBT, and a Random Forest Regressor (RFR) as base learners and MARS as a meta-learner for total organic carbon (TOC) prediction; they were able to generate more accurate and reliable TOC predictions than other single models. Wang et al. [33] also proposed a stacking machine learning model with ten individual models as its base models to predict E_g_ for 3896 inorganic compounds in E-AFLOW, achieving the best performance among other individual models evaluated by RMSE, MAE, MAPE, and R^2^ with 5-fold cross-validation, which demonstrates the excellent performance of the stacking approach to E_g_ prediction.

Chen et al. [34] applied six individual ML models for estimating the changes in mechanical properties in coals under CO_2_, including ANN, SVR, K-nearest neighbors (KNN), RFR and GBT, and then compared their performance with the ensemble stacking model with all six individual ML models as base models and linear regression as its meta-learner. The experimental result proved stacking model can perform better than other ML models. Deng et al. [35] developed a physics-informed machine learning (PIML) framework to model the creep-fatigue interaction behavior of a Ni-based superalloy, which also proves stacking ensemble learning algorithm can perform better, and they clearly pointed out that the assessment and selection of potential base models are critical for the proposed stacking model. However, all of these reported works only indicate that the performances of stacking models can be superior to those of single models used as the base models of the stacking models. None of them have discussed the performances of different stacking models with different combinations of base models and meta-learners, as well as the optimization methods for obtaining the most optimal stacking model. And few studies have been conducted stacking models with optimal methods for predicting properties of halide double perovskite, which has play import role in various fields.

It is meaningful to find out an effective optimization method to obtain the optimal stacking model, which simultaneously considers three indicators during the optimization process for the stacking model, including RMSE, MAE and R^2^. Nondominated sorting genetic algorithm (NSGA-II), proposed by Kalyanmoy Deb et al. [36], has become one of the most famous and widely used multi-objective evolutionary algorithms (MOEAs). Lee et al. [37] propose a computational strategy for perovskite discovery with few computing resources, considering both band gap and effective mass. Etghani et al. [38] employed NSGA-II for optimizing the process of perovskite solar cells with optimum conditions with multiple parameters to achieve PVSCs with high efficiency. However, the optimization results of the NSGA-II algorithm are presented as Pareto-optimal solution sets, which still need further sorting and optimization. TOPSIS is an ideal solution approximation ranking method based on distance, combined with the entropy weight method to achieve objective weight allocation, which has been widely applied in various fields [39,40,41], including the selection of ML models with optimal performance [42,43].

In this study, we proposed a method integrating TOPSIS with NSGA-II (TOPSIS- NSGA-II) to obtain the best combination of base models and meta-learner for constructing the optimal stacking regression model, which simultaneously considered four regression performance metrics(MSE, RMSE, MAE and R^2^) and the number of base models in the stacking models (N_base_) during the optimization process, achieving more efficient and reasonable decisions in multi-objective optimization for stacking model optimization. And the proposed method was utilized to predict the E_g_ and ΔH_f_ for 540 compounds of lead-free double halide perovskite with a feature set obtained from high-throughput calculations based on DFT in previous work [10]. The main contributions of our study are as follows:

(1) Construction of a new feature set easily obtained from the periodic table as input for ML models and utilization of Shapley Additive exPlanations (SHAP) in feature selection engineering for predicting E_g_ and ΔH_f_ of halide double perovskites were implemented.
(2) A method integrated NSGA-II and TOPSIS for stacking regression model optimization, simultaneously considering four regression metrics (MSE, MAE, RMSE and R^2^) and the number of base models constructed in the stacking models in the Pareto front was proposed.
(3) The optimal stacking regression model with high predicting accuracy was validated by a new dataset, providing guidance for discovering potential compounds for solar cells from a large quantity of materials.

**Figure 1:**
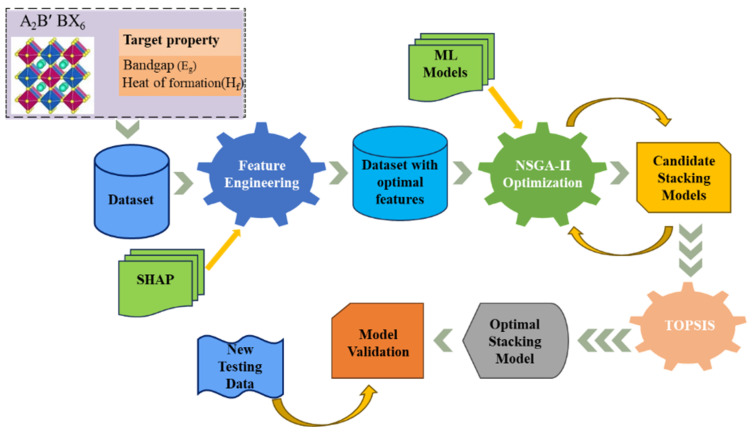
The overall workflow of the proposed method.

The remainder of this paper is organized as follows. Section 2 presents the methodology, including feature selection using SHAP (Section 2.1), model evaluation metrics (Section 2.2), stacking model optimization using NSGA-II (Section 2.3), and optimal model selection using TOPSIS (Section 2.4). Section 3 describes the experimental setup, covering the dataset (Section 3.1), input features and feature selection (Section 3.2), and the configuration of stacking ensemble regression models (Section 3.3). Section 4 provides the results and discussion, including performance comparisons between single and stacking models (Section 4.1), stacking models with different base model combinations (Section 4.2), stacking model optimization using NSGA-II and TOPSIS (Section 4.3), and model validation on a completely new test dataset (Section 4.4). Finally, Section 5 concludes the study with a summary of key findings and its limitations. And the optimal stacking model selected from the Pareto solutions by TOPSIS was validated on a new test dataset. Finally, a brief conclusion is given. The overall workflow of the proposed method is shown in Figure 1.

## 2. Methodology

### 2.1. Feature Selection with Shapley Additive exPlanations (SHAP)

SHAP proposed by Lundberg et al. [44], has been a popular tool used to interpret ML models with Shapley values in material science [45,46,47], including perovskites [48,49,50]. Unlike traditional feature selection methods [51,52,53], the SHAP method can deal with two strongly correlated features [10], and feature importance evaluated by SHAP value can satisfy consistency for each prediction process [4]. And the SHAP value can be used as a reliable property for feature attribution value comparison, making comparisons meaningful [54].

To identify the most predictive features for halide double perovskite properties, we applied the SHAP method following the approach established in our prior work [49]. As described in [49], SHAP is an additive feature attribution approach that decomposes the output of a ML model into the sum of contributions from each input feature, and it is approximated by a linear explanation model g formulated in Equation (1):

(1) $$g\left(z^{\prime }\right)=\;\varphi_{0}+\sum_{i=1}^{M}\varphi_{i}{z^{\prime }}_{i}$$

where $z\prime \in \{0,1{\}}^{M}$, indicates whether a feature is included (${z\prime }_{i}=1$) or excluded (${z\prime }_{i}=0$) from the model, *M* is the total number of input features, $\varphi_{0\;}$ represents the expected value when all inputs are missing, and $\varphi_{i}$ is the contribution value of a given feature *i* to the model presented in Equation (2).

(2) $$\varphi_{i}=\underset{S\subseteq F\setminus \left\{i\right\}}{\sum }\frac{\left|S\right|!\left(\left|F\right|-\left|S\right|-1\right)!}{\left|F\right|!}\left[f_{S\cup \left\{i\right\}}\left(x_{S\cup \left\{i\right\}}\right)-f_{S}\left(x_{S}\right)\right]$$

where F is the set of all features, $x_{S}$ represents the values of the input features in the set *S*. f is the original prediction model to be explained.

**Figure 2:**
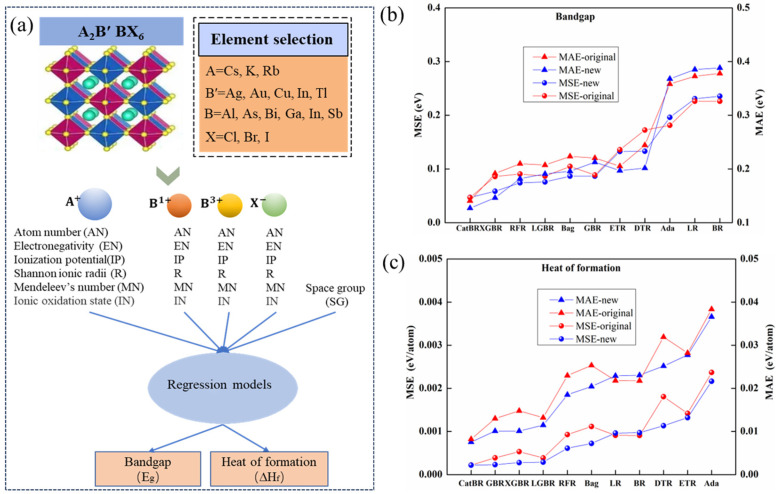
(**a**) The proposed new feature set for predicting bandgap and heat of formation. (**b**) Performance comparison between the original feature set in Ref. [10] and the proposed new feature set was measured by MAE and MSE from the averaged values of the testing set under 40 runs of five-fold cross-validation based on different regression models for predicting (**b**) bandgap and (**c**) heat of formation, respectively.

**Figure 3:**
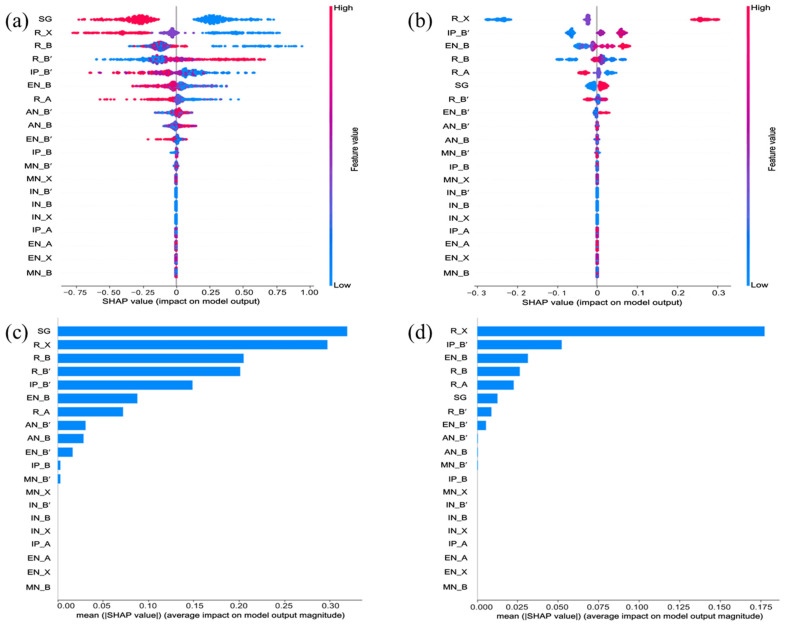
The summarized Sharpley values for the top 20 most important features presented with (**a**,**b**) global feature importance and (**c**,**d**) local explanation summary based on the XGBR model for bandgap (**a**,**c**) and heat of formation (**b**,**d**), respectively.

In this study, SHAP values were computed based on the optimal stacking models for E_g_ and ΔH_f_ prediction. Features (shown in Figure 2a) were ranked based on their feature importance values measured by their SHAP values, and the selected features were retained for subsequent experiments. The global feature importance rankings and local SHAP are visualized in Figure 3a–d.

### 2.2. Model Evaluation

The performance of the regression models was evaluated using four metrics: mean squared error (MSE), root mean squared error (RMSE), mean absolute error (MAE), and coefficient of determination(R^2^). These metrics are defined in Equations (3)–(6), respectively.

(3) $$MSE=\frac{1}{n}{\sum_{i=1}^{n}\left({\hat{y}}_{i}-y_{i}\right)}^{2}$$

(4) $$RMSE=\sqrt{\frac{1}{n}{\sum_{i=1}^{n}\left({\hat{y}}_{i}-y_{i}\right)}^{2}}$$

(5) $$MAE=\frac{1}{n}{\sum_{i=1}^{n}\left|y_{i}-{\hat{y}}_{i}\right|}^{2}$$

(6) $$R^{2}=1-\frac{{\sum_{i=1}^{n}\left|y_{i}-{\hat{y}}_{i}\right|}^{2}}{{\sum_{i=1}^{n}\left|y_{i}-\bar{y}\right|}^{2}}$$

where *n* is the number of samples, $\bar{y}$ is the averaged value of actual values, ${\hat{y}}_{i}$ and $y_{i}\;$represent the predicted value and the actual value of the $i_{th}$ sample, respectively. The regression performance of the models was evaluated using MSE, RMSE, MAE, and R^2^. These metrics served as the optimization objectives for the NSGA-II algorithm to identify the optimal meta-learner and combination of base models for constructing an ensemble stacking model with high performance. NSGA-II generates a Pareto front of non-dominated solutions, from which the final solution was selected using the Technique for Order Preference by Similarity to Ideal Solution (TOPSIS).

### 2.3. NSGA-II for Stacking Model Optimization with

The optimization objectives of NSGA-II are defined with the averaged values of MSE, RMSE, MAE, and R^2^, which are obtained from the stacking models through 40 iterations of five-fold cross-validation, along with the number of base models (N_base_) contained in each stacking model. Accordingly, the five optimization objectives of NSGA-II are expressed as:

(7) $$\left.\begin{array}{c} \;\;\;\;\;\;\;\;\;\;\;\;\;\;\;\;\;\;\;\;\;\;\;\;\;Minimize\;\;\;\;{\;\;f}_{1}=\bar{MSE}=\frac{1}{40}\sum_{i=1}^{40}{MSE}_{i}\;\;\;\\ \;\;\;\;\;\;\;\;\;\;\;\;\;\;\;\;\;\;\;\;{\;\;\;\;\;\;\;\;Minimize\;\;\;\;\;\;f}_{2}=\bar{RMSE}=\frac{1}{40}\sum_{i=1}^{40}{RMSE}_{i}\\ \;\;\;\;\;\;\;\;\;\;\;\;\;\;\;\;\;\;\;\;\;\;\;\;\;Minimize\;\;\;\;\;\;f_{3}=\bar{MAE}=\frac{1}{40}\sum_{i=1}^{40}{RMSE}_{i}\\ \;\;\;\;\;\;\;\;\;\;\;\;\;\;\;\;\;\;\;\;\;\;\;\;\;Maximize\;\;\;\;\;f_{4}=\bar{\;R^{2}}=\frac{1}{40}\sum_{i=1}^{40}{\left(R^{2}\right)}_{i}\;\;\;\;\;\;\;\\ \;Minimize\;\;\;\;\;\;f_{5}=N_{base}\;\;\;\;\;\;\;\;\; \end{array}\right\}$$

The output of NSGA-II is a Pareto front, which contains a set of candidate solutions consisting of different stacking models. And it is difficult to directly determine the optimal solution.

### 2.4. TOPSIS for Optimal Stacking Model Selection

To effectively select the optimal stacking model for the candidate solutions in the Pareto front generated by NSGA-II, the entropy-weighted TOPSIS method was employed [55]. The decision criteria consisted of N_base_ in the stacking ensemble, along with the average values of MSE, RMSE, MAE, and *R*^2^. Among these, N_base_, MSE, RMSE, and MAE were designated as cost criteria (lower is better), while *R*^2^ was designated as a benefit criterion (higher is better). The implementation strictly adhered to the standard TOPSIS procedure detailed in [55], and the model with the maximum relative closeness to the ideal solution was selected as the optimal solution for constructing the optimal stacking ensemble model, which defines the optimal meta-learner and base model combination, thereby constructing the final stacking ensemble model.

Assuming that the Pareto front comprises *m* candidate stacking models, and NSGA-II is formulated with *n* optimization objectives. As illustrated in [55], the decision matrix is constructed as follows:

(8) $$X=\left[\begin{array}{cccc} x_{11} & x_{12} & \cdots & x_{1n}\\ x_{21} & x_{22} & \cdots & x_{2n}\\ \vdots & \vdots & \ddots & \vdots \\ x_{m1} & x_{m2} & \cdots & x_{mn} \end{array}\right]$$

where $x_{ij}$ denotes the value of the *i*-th model with respect to the *j*-th objective. And the normalized decision matrix Z = ($z_{ij}$)*_m_*_×*n*_ can be constructed from matrix X = ($x_{ij}$)*_m_*_×*n*_ using Equation (9):

(9) $$z_{ij}=\frac{x_{ij}^{\prime }}{\sqrt{\sum_{i=1}^{m}{\left(x_{ij}^{\prime }\right)}^{2}}}$$

The weights of the objectives are calculated with the entropy weight method. Specifically, the weight $w_{j}$ of the *j*-th objective is derived from the proportion $p_{ij}$ of the *i*-th model with respect to the *j*-th objective and the information entropy $e_{j}\;$ of the *j*-th objective. They can be determined by Equation (10) as follows:

(10) $$p_{ij}=\frac{z_{ij}}{\sum_{i=1}^{m}z_{ij}},\;{\;e}_{j}=-\frac{1}{lnm}\sum_{i=1}^{m}p_{ij}ln\left(p_{ij}\right),\;{\;\;w}_{j}=\frac{{1-e}_{j}}{\sum_{j=1}^{n}d_{j}}$$

Subsequently, the weighted normalized decision matrix V = (*v_ij_*)*_m_*_×*n*_ is constructed, where *v_ij_* = *w_j_*⋅*z_ij_*_,_ as presented below:

(11) $$V=Z\cdot W=\left[\begin{array}{cccc} w_{1}z_{11} & w_{2}z_{12} & \cdots & w_{n}z_{1n}\\ w_{1}z_{21} & w_{2}z_{22} & \cdots & w_{n}z_{2n}\\ \vdots & \vdots & \ddots & \vdots \\ w_{1}z_{m1} & w_{2}z_{m2} & \vdots & w_{n}z_{mn} \end{array}\right]$$

The positive ideal solution $V^{+}$ and the negative ideal solution $V^{-}$ were subsequently determined as the best and worst values across all candidate solutions for each objective, respectively. Specifically, $V^{+}=\left(v_{1}^{+},v_{2}^{+},\cdots ,v_{n}^{+}\right)$, where $v_{j}^{+}=max\left(v_{1j},v_{2j},\cdots ,v_{mj}\right)$. And $V^{-}=\left(v_{1}^{-},v_{2}^{-},\cdots ,v_{n}^{-}\right)$, where $v_{j}^{-}=min\left(v_{1j},v_{2j},\cdots ,v_{mj}\right)$. The Euclidean distances from each solution to the positive and negative ideal solutions, denoted as $D_{i}^{+}$ and $D_{i}^{-}$, respectively, were then calculated using Equations (12) and (13).

(12) $$D_{i}^{+}=\sqrt{\sum_{j=1}^{n}{\left(v_{ij}-v_{j}^{+}\right)}^{2}}$$

(13) $$D_{i}^{-}=\sqrt{\sum_{j=1}^{n}{\left(v_{ij}-v_{j}^{-}\right)}^{2}}$$

The closeness degree of each solution is calculated with $D_{i}^{+}$ and $D_{i}^{-}$ expressed in Equation (14):

(14) $$C_{i}=\frac{D_{i}^{-}}{D_{i}^{+}+D_{i}^{-}},\;C_{i}\;\in [0,\;1]$$

The solution in the Parato front with higher closeness indicates it has better performance. The candidate stacking models are then ranked in descending order of *C_i_*, and the model with the highest *C_i_* is selected as the optimal stacking model.

## 3. Experimental Data and Model Construction

### 3.1. Dataset

The dataset for ML models in this study comprised 540 compounds of Pb-free double halide perovskites in the Supporting Information of earlier work [10], which were generated by high-throughput calculations based on DFT with the crystal structure of A_2_B′ BX_6_. Each compound contains 32 features named the original feature set, and some of them are obtained from DFT, making it difficult to apply to a new dataset of halide double perovskites. The heat of formation (ΔH_f_) values in this dataset are taken directly from [7] and reported in eV/atom—a common unit in DFT calculations (1 eV/atom = 96.485 kJ/mol).To overcome this difficulty, we constructed a new feature set that can be easily collected from accessible existing accessible tables (e.g., periodic table, Shannon ionic radii table and Mendeleev number table), which are shown in Figure 2a.

### 3.2. Input Features and Feature Selection

#### 3.2.1. Original Feature Set and New Constructed Feature Set

The accuracy of ML models partly depends on the input features [10,17,18,56,57]. Each perovskite compound in the dataset can be described by both elemental and structural features [19]. For halide double halide perovskites with A_2_B′ BX_6_ structure, it is known that input features consisting of Shannon ionic radii, atomic number, Mendeleev number, Pauling electronegativity, and ionization potential of A^+^, B′^+^, B^3+^, X^−^ site atoms have excellent performance on predicting thermodynamic stability of A_2_B′ BX_6_ double halide perovskites [49]. Since the space group (SG) information of the crystal is significant for bandgap and heat of formation, we continued to use SG from the dataset provided in the previous work [10], which only considers two crystal space groups, including cubic and orthorhombic. All input features used for prediction, named as the new feature set here, are shown in Figure 2a.

It is well known that the non-linear model generally achieves better performance than the linear model, and the tree-based model can provide more accurate predictions than other traditional models [58]. To compare performance of the newly proposed feature set with that of the original feature set, here we employed nine tree-base ML models for predicting bandgap (E_g_) and heat of information (ΔH_f_) based on original feature set and new proposed feature set, which includes Cat Boosting Regressor (CatBR) [59,60], eXtreme Gradient Boosting Regression (XGBR) [61], Random Forest Regressor (RFR) [62], Bagging Regressor (Bag) [63], Extra-Tree Regressor (ETR) [64], Gradient-Boosting Regressor (GBR) [60], Light Gradient Boosting Machine Regression (LGBR) [65], Decision Tree Regressor (DTR) [66], and AdaBoost (Ada) [67]. Together with another two simpler models, including Linear Regression (LR) and Bayesian Ridge (BR), simpler models have better ability to prevent overfitting [30]. The prediction results for E_g_ and ΔH_f_ from eleven individual regression models based on different feature sets can also be presented in Figure 2b and Figure 2c, respectively.

As shown in Figure 2b,c, most of the regression models with the new feature set can reach better performance for both E_g_ and ΔH_f_, in terms of averaged MAE and averaged MSE on the testing set via five-fold cross-validation repeated 40 times with different random states for dataset splitting. For E_g_ prediction (Figure 2b), there are notable differences in MSE and MAE between the original feature set and the new feature set. Taking CatBR, the best-performing model, as an example, the MSE and MAE based on the original feature set are 0.0470 eV and 0.1407 eV, respectively, while those based on the new proposed feature set were 0.0468 eV and 0.1272 eV, respectively. For ΔH_f_ prediction (Figure 2c), although the error gaps between the two feature sets are narrower, the new feature set still yielded improvements: the MAE decreased from 0.008217 eV/atom to 0.007549 eV/atom, and the MSE decreased from 0.000221 eV/atom to 0.000219 eV/atom. These results consistently demonstrate the superiority of the new feature set over the original one. Therefore, all subsequent experiments and discussions in this study are based on the proposed new feature set.

#### 3.2.2. Feature Selection by SHAP

In this study, we employed the SHAP value to evaluate the importance of features in the original feature set and the new feature set, respectively. The top 20 features ranked by SHAP values of the new feature set to predict E_g_ and ΔH_f_ are shown in Figure 3.

As shown in Figure 3, the top five features for E_g_ prediction are space group (SG) of crystal, R_X, R_B, R_B’ and IP_B’, while those for ΔH_f_ prediction are R_X, IP_B’, EN_B, R_B and R_A. Notably, SG ranks as the most important feature for E_g_ prediction but drops to sixth place for ΔH_f_ prediction. In contrast, several features—IN_A, IN_B’, IN_B and IN_X exhibit no importance for either target property, as they take constant values across all compounds in the dataset. Furthermore, SG emerges as the most import features for predicting E_g_ for halide double perovskites. In contrast to predicting ΔH_f_, the Shannon ionic radii of halogen anion (R_X) attain the highest importance score, followed by the ionization potential for B’^+^ (IP_B’) and electronegativity for B^3+^ (EN_B).

**Figure 4:**
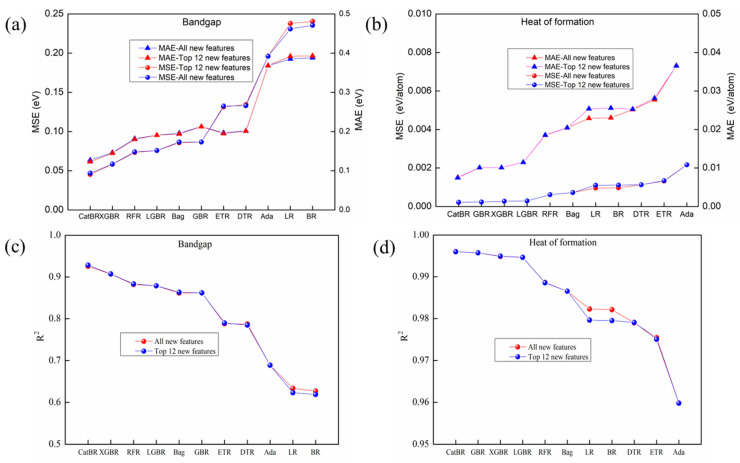
Predicting results of different regression models with the top 12 features selected by SHAP value and all 25 features as input, (**a**,**c**) for E_g_ prediction, (**b**,**d**) for ΔH_f_ prediction.

The prediction results, shown in Figure 4, show almost no degradation in performance for either E_g_ or ΔH_f_ prediction when using the top 12 features selected by SHAP values compared to using all 25 features. This finding provides strong evidence that SHAP values serve as an effective method for feature selection.

Furthermore, except for the input features, the abilities of different ML models are different, with a significant influence on the prediction results. That ML model with higher prediction for E_g_ and ΔH_f_ can greatly improve the capabilities of screening the most potential stable double halide perovskite from large chemical space for suitable applications. It is necessary to explore more accurate, effective hybrid models to achieve this goal. Since it has been proven that ensemble stacking models can generate better predictive performances, which has been widely used in various applications [28,29,30,68,69]. Therefore, we further employed an ensemble stacking model for predicting E_g_ and ΔH_f_ of halide double perovskites in the following sections.

### 3.3. Stacking Ensemble Regression Model

**Figure 5:**
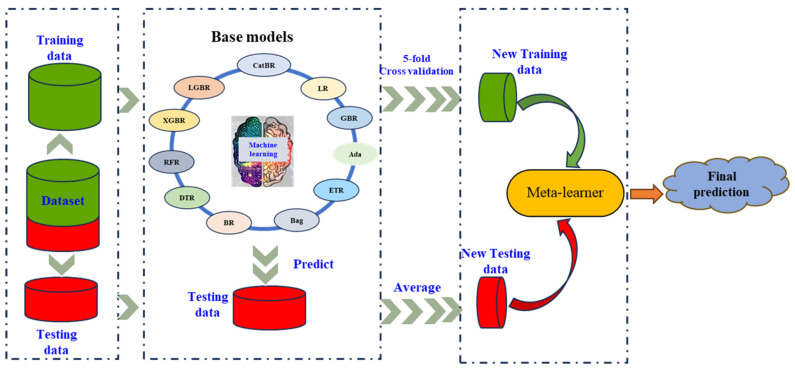
The workflow of the stacking model with 5-fold cross-validation for predicting E_g_ and ΔH_f_ for halide double perovskite.

Ensemble stacking is an advanced meta-learning algorithm designed to enhance predictive performance by combining multiple heterogeneous regression models, which consists of two layers. In the first layer, a diverse set of base models (e.g., CatBR, XGBR, RFR, LGBR) is trained independently on the original dataset to capture complementary patterns and relationships within the data. The predictions generated by these base models are then used as input features for the meta-learner in the second layer. Except for 11 models mentioned above, including CatBR, XGBR, RFR, LGBR, Bag, GBR, ETR, DTR, Ada, LR, BR, SVR model with the kernel of Radial Basis Function (SVR) usually performs well in the regression case, and it has also been widely used in perovskite material [70,71,72]. Hence, we also applied SVR in this work. In order to facilitate use and modeling, default parameters are used for both the single model and the combined model [73]. All single regression models used in this study are listed in Table 1. These single models have become candidate models for base models and a meta-learner used to construct a stacking model. The stacking model optimization is used to select the optimal combination of base models and a meta-learner to construct the optimal stacking model for higher prediction accuracy. The workflow of stacking model optimization with 5-fold cross-validation for predicting E_g_ and ΔH_f_ for halide double perovskite is shown in Figure 5.

In Figure 5, the dataset with input features optimized by the SHAP value employed in feature engineering was randomly split into two parts, including a training dataset and a testing dataset, both of which consisted of input features (viz. training data and testing data) and outputs (viz. training data label and testing data label). The training data was used to train twelve base models of the stacking model under 5-fold cross-validation to avoid overfitting [30]. The outputs of single models were used to construct new features from new training data as input to train the meta-learner, and the well-trained meta-learner was applied to predict new testing data with new features consisting of the averaged prediction results of the testing data. The final prediction was generated by the trained meta-leaner. And performances of different stacking models were measured by four metrics discussed in Equations (3)–(6) in Section 2.2.

## 4. Results and Discussion

In our study, all single ML models were implemented using the scikit-learn library in Python [74] (version 3.9.5). The test platform was a laptop equipped with Intel (R) Core (TM) i7-1165G7 CPU and 16G RAM. It also needs to be noted that all the results discussed below were measured by the averaged MAE, MSE and R^2^ according to 40 runs of 5-fold cross validation with 80% as training set and the rest of dataset as the testing set based on the 40 different random state, taking top 12 features selected by SHAP value as input, which were showed in Figure 4a for E_g_ prediction and Figure 4b for ΔH_f_ prediction.

### 4.1. Performance Comparisons Between Single Models and Stacking Models

**Figure 6:**
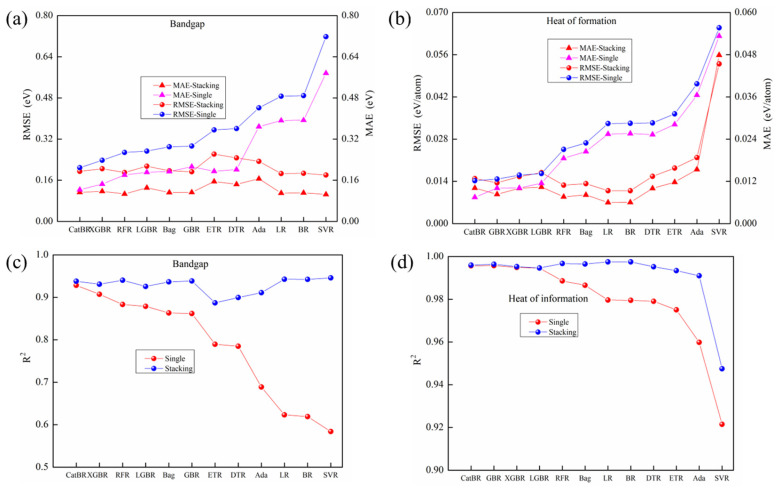
Performance comparison between 11 single models (CatBR, XGBR, RFR, LGBR Bag, GBR, ETR, DTR, Ada, LR and BR and stacking models with 11 single models as base models with different meta-learners. (**a**,**c**) for E_g_ prediction, (**b**,**d**) for ΔH_f_ prediction.

The results of regression models were averaged from 40 runs of 5-fold cross-validation for twelve single models (CatBR, XGBR, RFR, LGBR Bag, GBR, ETR, DTR, Ada, LR, BR and SVR) and twelve stacking models with twelve single models as base models with different meta-learners, which are marked on the horizontal axis shown in Figure 6. CatBR model yields the lowest values of MAE and RMSE among all the single models for both E_g_ (MAE with 0.1230 eV, RMSE with 0.2091 eV and R^2^ with 0.9282) and ΔH_f_ (MAE with 0.0075 eV/atom, RMSE with 0.0142 eV/atom and R^2^ with 0.9957), which is much lower than that reported in [10] calculated by the GBR model with 32 features obtained from DFT, with an averaged RMSE of 0.223 eV for E_g_ prediction, and an averaged RMSE of 0.021 eV/atom for ΔH_f_ prediction, achieving an improvement with 6.65% and 47.89%, respectively. However, they are inferior to almost all stacking models except for the stacking model with the LGBR model as the meta-learner, with slightly worse RMSE and R^2^ for ΔH_f_ prediction.

Furthermore, although the difference between different stacking models lies only in the meta model, there are significant differences in the performance of different stacking models. For E_g_ prediction, the stacking models with SVR as meta-learner can achieve the best performance in E_g_ prediction with the smallest MAE (0.1050 eV) and RMSE (0.1803 eV), highest R^2^ (0.9459), while the stacking model with the worst performance is the one with ETR as its meta-learner, with the highest MAE (0.1551 eV) and RMSE (0.2614 eV), lowest R^2^ (0.8871). Compared to CatBR, the best single model, the improvement percentage in RMSE and MAE of the stacking model with SVR as the meta-learner can reach 13.76% and 14.62%, while the improvement percentage in R^2^ is only 1.91%. More details concerning the performance comparison in E_g_ prediction can be seen in Table 2.

For ΔH_f_ prediction, stacking models with LR as the meta-learner can achieve the best performance with the smallest MAE (0.0060 eV/atom) and RMSE (0.0109 eV/atom) and the highest R^2^ (0.9975). Meanwhile, the worst stacking model is the one with Ada as its meta-learner, with the highest MAE (0.01569 eV/atom) and RMSE (0.02223 eV/atom), lowest R^2^ (0.99074). Compared to the best single model CatBR, the improvement percentage in RMSE and MAE to the stacking model with LR as the meta-learner can reach 23.35% and 19.79%, while the improvement percentage in R^2^ is only 0.17%. More details concerning the performance comparison in ΔH_f_ prediction can be seen in Table 3.

In summary, in both E_g_ prediction and ΔH_f_ prediction, the selection of the meta-learner for stacking models has a significant impact on the prediction performances of the stacking model. Although CatBR can achieve the best performance in both E_g_ and ΔH_f_ prediction among other single models, when using CatBR as the meta-learner for the stacking model in E_g_ prediction, its predictive performance is not as good as the stacking models with RFR, BR, LR, and SVR as the meta-models, respectively. This phenomenon also exists in ΔH_f_ prediction; when CatBR is used as the meta-learner of the stacking model, its predictive performance is not as good as the stacking models with RFR, BR, LR, and Bag as the meta-learners, respectively.

Furthermore, the predictive performance of the best stacking model with 12 single models as its base models is significantly improved compared to the best single model. It is still necessary to explore whether the combination of base models has an impact on predictive performance.

### 4.2. Stacking Model with Different Combinations of Base Models

**Figure 7:**
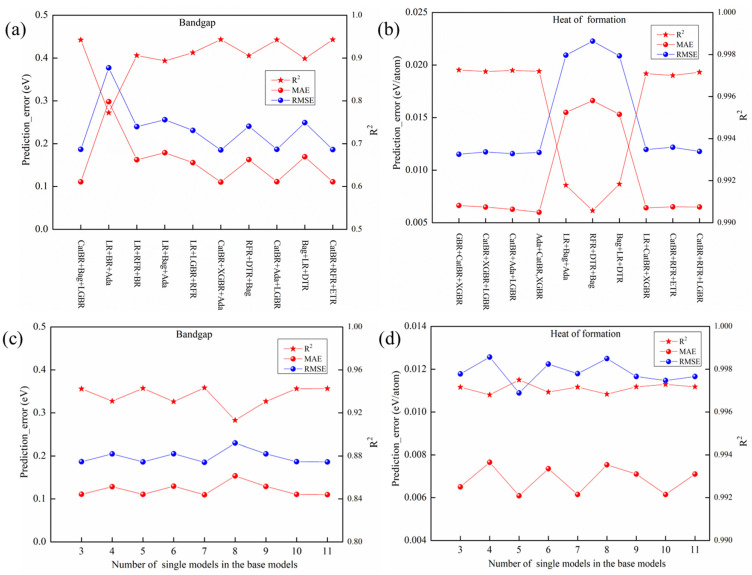
Performances of stacking model with LR as meta-learner and different combinations of base models randomly selected from twelve single models (CatBR, XGBR, RFR, LGBR, Bag, GBR, ETR, DTR, Ada, LR and BR). Base model combinations with three different single models for (**a**) E_g_ prediction and (**b**) ΔH_f_ prediction. Base model combinations with three to eleven single models for (**c**) E_g_ prediction and (**d**) ΔH_f_ prediction.

It can draw conclusions from Figure 7, which illustrates that the performances of stacking models with the same base model combination but different meta-learners vary significantly. And it clearly points out that the stacking model with twelve single models as base model and SVR as meta-learner can achieve the best performance in E_g_ prediction, while the stacking model with twelve single models as base model and LR as meta-learner can achieve the best performance in ΔH_f_ prediction. Furthermore, it is essential to investigate whether stacking models with different combinations of base models exhibit performance differences, providing guidance for subsequent optimization of stacking models.

Figure 7a illustrates that the stacking models for E_g_ prediction with the same number of single models, while different combinations as base models also perform differently, and all of them take SVR as their meta-learner. As the number of single models used to make up the base model of the stacking models increases, the regression performances of the stacking models do not improve accordingly. The performance of the stacking models formed by the same number of base models and meta models varies depending on the combination of base models. And there are significant differences in the prediction performance of the stacking model with SVR as its meta-learner and the same number of single models, while different combinations as its base models, the value of RMSE can be varied from 0.1788 eV to 0.3694 eV, the value of MAE can be varied from 0.1039 eV to 0.2739 eV, and the value of R^2^ can be varied from 0.7807 to 0.9468. The stacking model, which employs only CatBoost, Bagging, and LightGBM (marked as CatBR + Bag + LGBR) as base learners with SVR as meta-learner, achieved slightly superior performance to the full ensemble of twelve base models. Specifically, it reduced the MAE from 0.1050 eV to 0.1039 eV and the RMSE from 0.1803 eV to 0.1788 eV, while increasing R^2^ from 0.9459 to 0.9468. Critically, this near-equivalent performance was attained using only three base models instead of twelve. This dramatic reduction in model complexity, coupled with the observed marginal performance gains, underscores the necessity and potential value of systematically optimizing the combination of both base learners and the meta-learner in stacking frameworks.

For ΔH_f_ prediction (Figure 7b), a contrasting yet insightful trend is observed: a stacking model with only three base models (marked as GBR + CatBR + XGBR) and an LR meta-learner delivers nearly equivalent performance (MAE = 0.00664 eV/atom, RMSE = 0.01151 eV/atom, R^2^ = 0.99727) to the all twelve-model ensemble (MAE = 0.00600 eV/atom, RMSE = 0.01090 eV/atom, R^2^ = 0.99750), despite a 75% reduction in base models. This demonstrates it needs further research into optimal base-model and meta-model composition strategies.

Furthermore, to systematically investigate the relationship between the number of base learners and the regression performance of the stacking ensemble, we constructed multiple stacking variants by incrementally increasing the count of constituent base models. For the E_g_ prediction task (Figure 7c), SVR served as the meta-learner, while Linear Regression (LR) was used for the ΔH_f_ prediction task (Figure 7d). The experimental results clearly demonstrate that predictive performance does not monotonically improve with an increase in the number of base models.

This trend is explicitly illustrated in Figure 7c,d. For the E_g_ prediction (Figure 7c), as the number of single models used to construct the base models of stacking ensembles increases—through random combinations of the single models listed in Table 1—the predictive performance of the stacking models does not improve correspondingly. Instead, it exhibits irregular fluctuations. Similarly, for the ΔH_f_ prediction (Figure 7d), increasing the ensemble size leads to fluctuating performance without a clear positive trend. The complete specifications of the base model combinations for each stacking configuration and their associated test set performance (based on 40 runs of 5-fold cross-validation) are provided in Supplementary Tables S1 and S2.

### 4.3. Stacking Model Optimization with NSGA-II +TOPSIS

Due to the limited combinations of base models considered, it is difficult to identify the optimal stacking model using conventional approaches. Therefore, a more convenient optimization method is required that can incorporate a broad range of combinations of base models and meta-models. In this study, NSGA-II implemented via the DEAP library was employed for stacking model optimization, which simultaneously considers the number of base models (N_base_) in stacking models and the averaged values of MSE, RMSE, MAE, and R^2^ through 40 iterations of five-fold cross-validation, and it yields optimal results in the form of a Pareto front containing multiple non-dominated solutions. Subsequently, TOPSIS was utilized to select the best solution from the Pareto front, and the optimal stacking model was identified based on the closeness degree calculated using Equation (14).

The NSGA-II algorithm is executed with a specific set of parameters to evolve a population of solutions, as shown in Table 4. The population is initialized with 20 individuals, and the algorithm runs for 20 generations to iteratively improve this population. The evolutionary process is guided by a crossover probability of 0.8, and the mutation probability is set to 0.2. The performance of the individual stacking model is evaluated with the averaged MSE, RMSE, MAE and R^2^, which are obtained from 40 runs of 5-fold validation, and they are treated as fitness values during the evaluation process. After the evolutionary process completes, the best-performing individual is selected from the final population, decoded into its constituent base models and meta-model. The NSGA-II algorithm is employed to identify a set of Pareto-optimal solutions, known as the Pareto front, rather than a single optimum.

**Figure 8:**
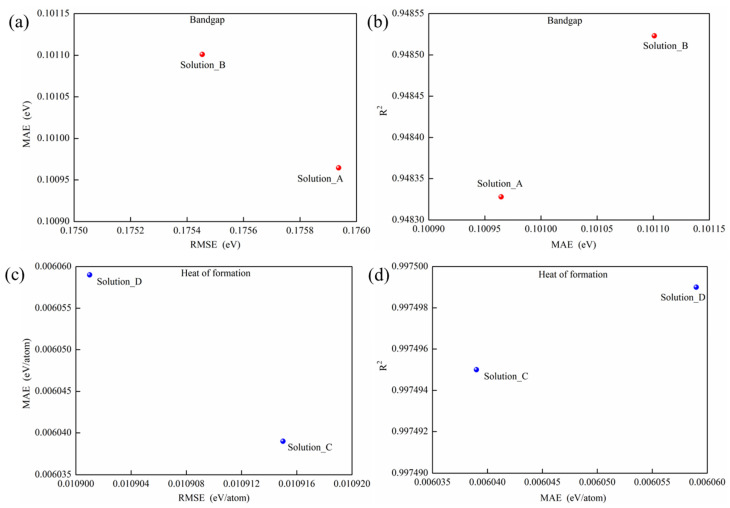
Pareto-optimal solutions from NSGA-II for optimizing the stacking model evaluated with averaged MAE, RMSE and R^2^ over 40 runs of 5-fold cross-validation. (**a**) MAE vs. RMSE and (**b**) R^2^ vs. MAE for predicting E_g_; (**c**) MAE vs. RMSE and (**d**) R^2^ vs. MAE for predicting ΔH_f_.

It is well known that the optimization results obtained from the NSGA-II algorithm constitute a set of Pareto-optimal solutions rather than a single optimum. In this study, separate NSGA-II runs were configured with 20 generations to optimize the stacking ensembles for bandgap (E_g_) and heat of formation (ΔH_f_) prediction, respectively. Each optimization yielded a set of 20 Pareto-optimal solutions, and the consistent convergence behavior observed in the NSGA-II optimizations is illustrated in Figure 8.

As shown in Figure 8, the Pareto solutions for both E_g_ and ΔH_f_ prediction are heavily concentrated on two stacking models. For E_g_ prediction, the averaged RMSE of Solution_B is lower than that of Solution_A, whereas the MAE of Solution_A is lower than that of Solution_B (Figure 8a), and the R^2^ of Solution_B is higher than that of Solution_A (Figure 8b). For ΔH_f_ prediction, the averaged RMSE of Solution_D is lower than that of Solution_C, whereas the MAE of Solution_C is lower than that of Solution_D (Figure 8c), and the R^2^ of Solution_D is higher than that of Solution_C (Figure 8d). More detailed information on the Pareto solutions is presented in Table 5.

As shown in Figure 8 and Table 5, it is difficult to distinguish which solution is the optimal one. Thereby, TOPSIS is employed for selecting the optimal stacking model from the Pareto solutions by calculating the closeness degree (C_I) of each solution, which simultaneously takes five metrics (averaged MSE, RMSE, MAE, R^2^ and the number of base models) into consideration, and considers the number of individual models that comprise the base model of the stacking model. And the weights of them can be calculated with Equation (10), respectively. And the results calculated by TOPSIS can also be seen in Table 5.

As summarized in Table 5a, the optimal stacking model selected by TOPSIS for E_g_ prediction is the Solution_B with a closeness degree of 0.6441, which consists of four base models—CatBR, RFR, GBR, ETR, and employs SVR with a radial basis function (RBF) kernel as the meta-learner. The optimized model achieved the lowest averaged MSE of 0.0323 eV^2^, RMSE of 0.1755 eV, MAE of 0.1011 eV, and R^2^ of 0.9485. Compared to the best unoptimized stacking model (comprising 12 base models), the optimized model yielded improvements of 5.02%, 2.70%, 3.72% and 0.28% in MSE, RMSE, MAE and R^2^, respectively. Notably, the number of base models was substantially reduced from 12 to 4 after optimization. A detailed performance comparison between unoptimized and optimized stacking models for E_g_ prediction is presented in Table 6.

In terms of ΔH_f_ prediction in Table 5b, Solution_D was identified as the optimal stacking model by TOPSIS with a closeness degree of 0.9553. This model consists of six base models—CatBR, XGBR, Bag, GBR, Ada and LGBR and uses BR as the meta-learner. Compared to the unoptimized model, the optimized model achieved averaged values of 0.000136, 0.010901, 0.006059 and 0.997499 for MSE, RMSE, MAE and R^2^, respectively, exhibiting improvements of 0%, 0.06%, 0.8% and 0.0005% in the respective metrics. Furthermore, the number of base models decreased sharply from 12 to 6. Performance comparison of unoptimized and optimized stacking models for ΔH_f_ prediction is also summarized in Table 7.

**Figure 9:**
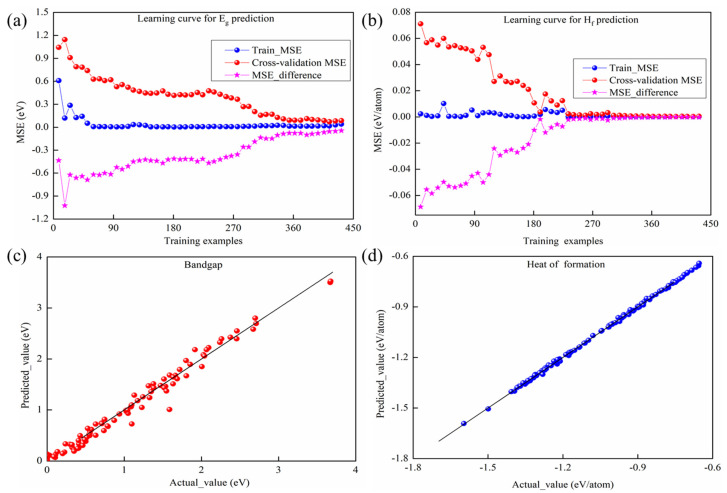
Performances of the optimal stacking models. (**a**) Learning curves for E_g_ prediction; (**b**) Learning curves for ΔH_f_ prediction; (**c**) Parity plot of predicted vs. actual E_g_ values; (**d**) Parity plot of predicted vs. actual ΔH_f_ values. In (**a**,**b**), Train-MSE, Cross-validation MSE, and their difference (MSE_difference, calculated as Train-MSE minus Cross-validation MSE) are shown. In (**c**,**d**), the ideal matchup between predicted and actual values is shown as the 45° dashed line.

In addition, it is necessary to verify whether an overfitting problem exists in the optimal stacking prediction models determined by learning curves [20]. Figure 9 illustrates the learning curves of the optimal stacking models for E_g_ prediction (Figure 9a) and for ΔH_f_ prediction (Figure 9b), depicting the relationship between the number of training samples and the proposed model’s performance. With the increase in training samples, both the Train-MSE and Cross-validation MSE show a decreasing trend and converge to their minimum values when the training size is approximate to 80%, and the gap between them narrows to near zero, as shown by the MSE_difference curve, confirming the proposed models’ strong generalization ability and high predictive accuracy without overfitting.

To further validate the predictive performance of the proposed stacking models, we compared their predicted values with the actual values for E_g_ and ΔH_f_, respectively. The comparison was based on 108 compounds randomly selected from the dataset, using the top 12 features from the new feature set. For E_g_ prediction, the optimal stacking model employed CatBR, RFR, GBR, and ETR as base models, with SVR (RBF kernel) as the meta-learner. The predicted results are presented in Figure 9c, which are highly consistent with the actual values. In the ΔH_f_ prediction, the predicted ΔH_f_ values from the optimal stacking model show excellent agreement with the actual values, as shown in Figure 9d. This model employed CatBR, XGBR, Bag, GBR, Ada and LGBR as base models and LR as the meta-learner. The small deviations observed across all test samples provide compelling evidence of the model’s strong generalization capability and high predictive accuracy.

### 4.4. Comparison of the Optimal Stacking Model Selected by TOPSIS Versus the Optimal Model Identified by Random Search

To further validate the effectiveness of the NSGA-II optimization combined with TOPSIS selection, we compared the optimal stacking model selected by TOPSIS against the optimal model identified by 400 random searches under the same evaluation budget (each model evaluated with 40 runs of 5-fold cross-validation). Table 8 presents the comparison results for both prediction targets, split into two sub-tables for clarity due to the different units (eV for E_g_, eV/atom for ΔH_f_).

Table 8a shows the results for E_g_ prediction. The TOPSIS-selected model achieves an RMSE of 0.1755 eV using only 4 base models (CatBoost, RandomForest, GradientBoosting, and ExtraTree) with SVR as the meta-learner. In contrast, the optimal model identified by random search achieves an RMSE of 0.1783 eV using 6 base models (CatBoost, XGBoost, GradientBoosting, ExtraTree, AdaBoost, and LinearRegression), also with SVR as the meta-learner. The TOPSIS-selected model yields relative improvements of 1.59% in RMSE, 2.47% in MSE, and 2.91% in MAE, while reducing the base model size by 33.33% (from 6 to 4 models).

Table 8b presents the results for ΔH_f_ prediction. The TOPSIS-selected model achieves an RMSE of 0.010901 eV/atom using 6 base models (CatBoost, Bagging, XGBoost, GradientBoosting, AdaBoost, and LightGBM) with Bayesian Ridge as the meta-learner. The optimal random search model achieves an RMSE of 0.011119 eV/atom using 7 base models (CatBoost, Bagging, GradientBoosting, ExtraTree, AdaBoost, LightGBM, and LinearRegression) with Bayesian Ridge as the meta-learner. The TOPSIS-selected model demonstrates relative improvements of 1.96% in RMSE, 3.49% in MSE, and 5.76% in MAE, with a 14.29% reduction in base model size (from 7 to 6 models).

To confirm that the proposed NSGA-II+TOPSIS method does not lead to performance degradation, we performed one-tailed paired t-tests comparing its RMSE values against those of the optimal random search model over 40 independent cross-validation runs. For E_g_ prediction (Table 8a), the test yields a *p*-value of 0.0603; for ΔH_f_ prediction (Table 8b), the *p*-value is 0.2791. Both *p*-values exceed the conventional significance level of 0.05, statistically confirming no performance degradation for either prediction target. These results demonstrate that NSGA-II with TOPSIS selection effectively identifies more compact stacking ensembles without sacrificing predictive performance.

### 4.5. Model Validation on Completely New Test Data

The training dataset contains three different A-site cations (viz. Cs, K, and Rb), five B1-site cations (viz. Tl, In, Ag, Au, and Cu) and six B2-site cations (viz. Al, Ga, Bi, In, Sb, and As). It appears on either B1- or B2-sites. The X-site has four different possible choices of F, Cl, Br, and I. And two kinds of the crystal group space were taken into consideration, which consisted of a total of 540 unique A_2_B′ BX_6_ compounds in the chemical space. Since all possible compounds in the chemical space are included in the training dataset, we needed to find other data for model validation.

To conform the predicting ability of our proposed hybrid stacking model, we applied it to 24 new compounds in cubic crystal space excluded from the training dataset, six compounds of which contained 3 completely new elements (viz. Na^+^, K^+^, and Rb^+^) in B-site never appeared in the training process, and their values used in the comparison for E_g_ and decomposition enthalpy (ΔH) were calculated by DFT obtained from the work of Zhao et al. [75], which contains 64 compounds, of which 40 already exist in the dataset used for stacking model optimization, and the remaining 24 compounds are used as a new dataset to validate the proposed optimal stacking model. The average predicted results were depicted in Table 9.

There are 2 out of 24 compounds (viz. Cs_2_NaBiBr_6_ with 0.7174 eV, and Cs_2_NaBiI_6_ with 0.7065 eV) in the classified criteria from Im [10] within the range of [0.3 eV, 0.8 eV] according to the predicted E_g_ from the proposed model, indicating that they are suitable to be material for solar cells, which is not in line with the results calculated by Zhao [75], where the classified criteria for solar cells are within the range of [0.8 eV, 2 eV]. It needs to be noted that they are all within the averaged MAE of 0.1011 eV obtained by our proposed method.

In addition, the stability is also another significant property for solar cells, and they can be evaluated to be stable with negative values of ΔH_f_ [10] or positive decomposition enthalpy [75]. From Table 9, it shows all of them are stable due to their negative values in predicting ΔH_f_. 4 out of 24 compounds are classified to be unstable according to the negative decomposition enthalpy (viz. Cs_2_KSbI_6_ with −3 meV/atom, Cs_2_KBiI_6_ with −4 meV/atom, Cs_2_RbSbI_6_ with −14 meV/atom, and Cs_2_RbBiI_6_ with −15 meV/atom), and the calculated values of decomposition enthalpy (ΔH) of Cs_2_KSbI_6_ and Cs_2_KBiI_6_ are very close to the stable criterions of positive decomposition enthalpy. These results proved that the proposed model can be an effective method for quickly predicting E_g_ and ΔH_f_ for halide double perovskites to identify compounds with elements appearing frequently in the training dataset, obtaining compounds with suitable E_g_ and ΔH_f_ for solar cells. However, it may probably not be effective for compounds with elements that are contained in the training dataset, which can lead to a conclusion consistent with that mentioned in [76]. It is worth noting that it took only a few seconds to predict all test compounds with the trained stacking model on the laptop with Intel(R) Core (TM) i7-1165G7 CPU.

The few discrepancies in Table 9 (2 for band gap, 4 for stability) are predominantly observed in compounds containing the new alkali metal ions (Na^+^, K^+^, Rb^+^), specifically Cs_2_NaBiBr_6_ and Cs_2_NaBiI_6_ for band gap and Cs_2_KSbI_6_, Cs_2_RbSbI_6_, Cs_2_KBiI_6_, and Cs_2_RbBiI_6_ for stability. These Bi/I-containing systems are particularly sensitive to DFT computational settings. Critically, as noted in Zhao et al. [75], their DFT calculations included spin–orbit coupling (SOC), whereas our training data from Im et al. [10] were computed using the PBE functional without SOC. It is well-established that SOC can reduce the band gaps of Bi/I-containing perovskites by 0.5–1.5 eV and alter their formation energies by 0.3–0.7 eV/atom. Consequently, the observed discrepancies are more likely attributable to these differences in DFT benchmarks rather than to a fundamental error in our model.

### 4.6. Generalizability to Other Perovskite Families

To evaluate the generalizability of our proposed feature set and stacking-based classification methodology, we applied them to the expanded double perovskite dataset compiled by Bartel et al. [51], which contains 918 A_2_B′BX_6_ materials including both halide and oxide compositions. This classification task enables a direct benchmark against Bartel‘s τ descriptor (91% accuracy).

As shown in Table 10, our proposed feature set combined with the optimal stacking configuration (CatBoost Classifier, Random Forest Classifier, Bagging Classifier, AdaBoost Classifier + CatBoost Classifier) identified by our framework—achieves superior performance, with an accuracy of 96.22% (±1.01%), an F1 score of 98.03% (±0.53%), and a precision of 96.89% (±0.83%) under 40 runs of 5-fold cross-validation, surpassing the 91% baseline.

These results demonstrate: (1) our feature set generalizes across perovskite families; (2) our feature selection and stacking optimization framework is versatile, effectively handling both regression and classification tasks; and (3) extension to Ruddlesden–Popper phases requires additional layer-specific descriptors (future work).

## 5. Conclusions

In this study, we aimed to developed a method integrated NSGA-II and TOPSIS for stacking regression model optimization, which simultaneously considered four regression metrics (MSE, MAE, RMSE and R^2^) and the number of base models constructed the stacking models as the five optimization targets, and it was utilized for the quantitative determination of E_g_ and ΔH_f_ of 540 Pb-free halide double perovskites with new constructed feature set obtained from easily accessible tables. The experimental results have demonstrated that the newly constructed feature set has better performance than the original feature set. It indicated that the SHAP value can be an effective tool for feature selection as well. Furthermore, it also proved that the ensemble stacking model can perform better than any individual ML model in both E_g_ and ΔH_f_ prediction with lower MSE and MAE for double halide perovskites with the top 12 features from the new feature set selection by SHAP values. The combination of NSGA-II and TOPSIS has performed well in the ensemble stacking model optimization. The stacking model optimized by our proposed method yields better predicting performance than the stacking regression models without optimization, with improvements in averaged MSE, RMSE, MAE and R^2^ with 5.02%, 2.70%, 3.72% and 0.28% in E_g_ prediction, respectively. And for ΔH_f_ prediction, as its predictive accuracy was inherently high, despite only a minor performance improvement after optimization, the number of base models for the stacking model dropped sharply from 12 to 6. Finally, the proposed models were validated on completely new test data. The limitations of our study are that the prediction performance of compounds in the testing dataset with elements not contained in the training dataset will be degraded. The training dataset should be extended to contain more elements consisting of compounds in the whole chemistry space to improve the predictive ability. In addition, multi-objective optimization should be introduced to take key properties of halide double perovskite into account simultaneously for screening suitable materials for solar cells more quickly and conveniently. These will be the focus of our research in the future.

## Figures and Tables

**Table 1:** Index for single ML models used as a meta-learner in hybrid stacking models to select the optimal model.

Model ID	Model Name	Note
1	CatBR	Gradient Boosting with categorical feature support
2	XGBR	Extreme Gradient Boosting Regressor
3	RFR	Random Forest Regressor
4	LGBR	Light Gradient Boosting Machine Regression
5	Bag	Bagging Regressor
6	GBR	Gradient Boosting Regressor
7	ETR	Extra-Tree Regressor
8	DTR	Decision Tree Regressor
9	Ada	AdaBoost Regressor
10	LR	Linear regressor
11	BR	Bayesian Ridge
12	SVR	SVR with RBF kernel

**Table 2:** Performance comparison between stacking models and single models in E_g_ prediction.

Stacking Models				Single Models			
Meta-Learner	RMSE (eV)	MAE (eV)	R^2^	Model Name	RMSE (eV)	MAE (eV)	R^2^
CatBR	0.1944	0.1131	0.9381	**CatBR**	**0.2091**	**0.1230**	**0.9282**
XGBR	0.2047	0.1173	0.9308	XGBR	0.2375	0.1450	0.9073
RFR	0.1903	0.1070	0.9402	RFR	0.2681	0.1801	0.8833
LGBR	0.2145	0.1307	0.9256	LGBR	0.2730	0.1909	0.8788
Bag	0.1965	0.1124	0.9364	Bag	0.2899	0.1939	0.8632
GBR	0.1932	0.1129	0.9384	GBR	0.2927	0.2126	0.8619
ETR	0.2614	0.1551	0.8871	ETR	0.3557	0.1949	0.7895
DTR	0.2469	0.1442	0.8997	DTR	0.3607	0.2012	0.7848
Ada	0.2334	0.1659	0.9110	Ada	0.4419	0.3684	0.6887
LR	0.1862	0.1102	0.9427	LR	0.4865	0.3920	0.6231
BR	0.1868	0.1108	0.9423	BR	0.4891	0.3933	0.6192
**SVR**	**0.1803**	**0.1050**	**0.9459**	SVR	0.7182	0.5759	0.5841

Notes: All stacking models consist of the 12 base models detailed in Table 1, with the meta-learner for each model listed in the “Meta-learner” column. The best-performing single model and the best-performing stacking model, along with their respective performance metrics, are highlighted in bold.

**Table 3:** Performance comparison between stacking models and single models in ΔH_f_ prediction.

Stacking Models				Single Models			
Meta-Learner	RMSE (eV/atom)	MAE (eV/atom)	R^2^	Model Name	RMSE (eV/atom)	MAE (eV/atom)	R^2^
CatBR	0.01460	0.00985	0.99598	**CatBR**	**0.01422**	**0.00748**	**0.99585**
GBR	0.01344	0.00830	0.99642	GBR	0.01475	0.01010	0.99572
XGBR	0.01543	0.00973	0.99536	XGBR	0.01608	0.01011	0.99493
LGBR	0.01827	0.01090	0.99370	LGBR	0.01662	0.01148	0.99464
RFR	0.01269	0.00761	0.99675	RFR	0.02460	0.01856	0.98857
Bag	0.01325	0.00807	0.99649	Bag	0.02672	0.02046	0.98654
**LR**	**0.01090**	**0.00600**	**0.99750**	LR	0.03314	0.02542	0.97962
BR	0.01092	0.00601	0.99749	BR	0.03324	0.02558	0.97950
DTR	0.01694	0.01018	0.99425	DTR	0.03336	0.02529	0.97907
ETR	0.01817	0.01150	0.99348	ETR	0.03639	0.02816	0.97506
Ada	0.02223	0.01569	0.99074	Ada	0.04637	0.03654	0.95982
SVR	0.05297	0.04787	0.94750	SVR	0.06494	0.05328	0.92149

Notes: All stacking models consist of the 12 base models detailed in Table 1, with the meta-learner for each model listed in the “Meta-learner” column. The best-performing single model and the best-performing stacking model, along with their respective performance metrics, are highlighted in bold.

**Table 4:** Parameters of NAGA-II for stacking model optimization in E_g_ and ΔH_f_ prediction.

Parameter	Value	Description
Population Size	20	Number of candidate stacking model configurations in each generation.
Generations	20	Total iterations of the evolutionary process.
Crossover Probability	0.8	Probability of performing crossover between two parent solutions.
Mutation Probability	0.2	The probability that an individual will undergo mutation.
Number of fitness evaluations	40 runs × 5-fold CV	Each stacking model evaluated by 40 repeated runs of five-fold cross-validation.
Fitness metrics	MSE, RMSE, MAE, R^2^, N_base_	Five regression metrics used as multi-objective optimization criteria.

**Table 5:** Performance of Pareto solutions and the optimal stacking model selected with TOPSIS. (**a**) Band gap (E_g_) prediction. (**b**) Heat of formation (ΔH_f_) prediction.

(**a**)									
**Pareto Solutions**	**Base_Model_Size**	**Base_Model**	**Meta-Learner**	**MSE (eV^2^)**	**RMSE (eV)**	**MAE (eV)**	**R^2^**	**C_I**	**Optimal Model**
Solution_A	4	CatBR, GBR, ETR, Ada	SVR	0.0324	0.1759	0.1010	0.9483	0.3559	N
Solution_B	4	CatBR, RFR, GBR, ETR	SVR	0.0323	0.1755	0.1011	0.9485	0.6441	Y
(**b**)									
**Pareto Solutions**	**Base_Model_Size**	**Base_Model**	**Meta-Learner**	**MSE (eV^2^/atom^2^)**	**RMSE (eV/atom)**	**MAE (eV/atom)**	**R^2^**	**C_I**	**Optimal Model**
Solution_C	8	CatBR, RFR, XGBR, Bag, GBR, ETR, Ada, LGBR	BR	0.000136	0.010915	0.006039	0.997495	0.0447	N
Solution_D	6	CatBR, Bag, XGBR, GBR, Ada, LGBR	BR	0.000136	0.010901	0.006059	0.997499	0.9553	Y

**Table 6:** Performance comparison of unoptimized and optimized stacking models for E_g_ prediction.

Metric	Best Stacking Model (Unoptimized)	Optimal Stacking Model (Optimized)	Improvement
MSE (eV^2^)	0.0340	0.0323	↓ 5.02%
RMSE (eV))	0.1803	0.1755	↓ 2.70%
MAE (eV)	0.1050	0.1011	↓ 3.72%
R^2^	0.9459	0.9485	↑ 0.28%
Base Model Size	12 base models	4 base models	↓ 66.7%
Meta-learner	SVR	SVR	—

Note: ↑ indicates improvement after optimization; ↓ indicates degradation after optimization; — indicates not applicable. The direction of improvement (↑ or ↓) is defined based on the desirability of each metric (e.g., for R^2^, ↑ is better; for RMSE, MSE, MAE and the Base Model Size, ↓ is better).

**Table 7:** Performance comparison of unoptimized and optimized stacking models in ΔH_f_ prediction.

Metric	Best Stacking Model (Unoptimized)	Optimal Stacking Model (Optimized)	Improvement
MSE (eV^2^/atom^2^)	0.000136	0.000136	0%
RMSE (eV/atom)	0.010907	0.010901	↓ 0.06%
MAE (eV/atom)	0.006011	0.006059	↓ 0.8%
R^2^	0.997494	0.997499	↑ 0.0005%
Base Model Size	12 base models	6 base models	↓ 50%
Meta-learner	LR	BR	—

Note: ↑ indicates improvement after optimization; ↓ indicates degradation after optimization; — indicates not applicable. The direction of improvement (↑ or ↓) is defined based on the desirability of each metric (e.g., for R^2^, ↑ is better; for RMSE, MSE, MAE and the Base Model Size, ↓ is better).

**Table 8:** Performance comparison between the optimal stacking model selected by TOPSIS and the best stacking model identified by 400 random searches. (**a**) Band gap (E_g_) prediction; (**b**) heat of formation (ΔH_f_) prediction.

(**a**)							
**Model**	**Base_Model_Size**	**Base_Model**	**Meta-Learner**	**MSE** **(eV^2^)**	**RMSE** **(eV)**	**MAE** **(eV)**	**R^2^**
TOPSIS-selected	4	CatBR, RFR, GBR, ETR	SVR	0.0323 (±0.0134)	0.1755 (±0.0375)	0.1011 (±0.0139)	0.9485 (±0.0223)
Optimal from 400 RS	6	CatBR, XGBR, LR, GBR, ETR, Ada,	SVR	0.0331 (±0.0135)	0.17834 (±0.0382)	0.1041 (±0.0142)	0.9472 (±0.0225)
Improvement (%)	+33.33%	—	—	+2.47%	+1.59%	+2.91%	+0.14%
(**b**)							
**Model**	**Base_Model_Size**	**Base_Model**	**Meta-Learner**	**MSE** **(eV^2^/atom^2^)**	**RMSE** **(eV/atom)**	**MAE** **(eV/atom)**	**R^2^**
TOPSIS-selected	6	CatBR, Bag, XGBR, GBR, Ada, LGBR	BR	0.000136 (±0.000111)	0.010901 (±0.004134)	0.006059 (±0.000932)	0.997499 (±0.002038)
Optimal from 400 RS	7	CatBR, Bag, GBR, ETR, Ada, LGBM, LR	BR	0.000141 (±0.000113)	0.011119 (±0.004181)	0.006430 (±0.000928)	0.997406 (±0.002081)
Improvement (%)	14.29%	—	—	+3.49%	+1.96%	+5.76%	+0.01%

Note: “Optimal from 400 RS” refers to the stacking model with the lowest RMSE among 400 randomly sampled combinations. Values in parentheses represent standard deviations over 40 independent cross-validation runs. One-tailed paired *t*-tests (TOPSIS-selected vs. Optimal from 400 RS) on RMSE values yield *p* = 0.0603 for E_g_ and *p* = 0.2791 for ΔH_f_, confirming no statistical degradation for either target. Improvement (%) = (RS − TOPSIS)/RS × 100% for MSE, RMSE, MAE; for R^2^, improvement = (TOPSIS − RS)/|RS| × 100%. Conversion: 1 eV/atom = 96.485 kJ/mol.

**Table 9:** Predicting results of E_g_ and ΔH_f_ for 24 new compounds by the proposed hybrid stacking model.

Nos.	Compounds	Predicted E_g_ (eV)	Classified with Predicted E_g_^a^	Classified with Calculated E_g_^b^	Predicted ΔH_f_ (_eV/atom_)	Stability Classified with Predicted ∆H_f_^a^	Stability Classified with Calculated ∆H^b^
1	Cs2NaSbBr6	1.2198	N	N	−0.7630	Y	Y
2	Cs2KSbBr6	0.9398	N	N	−0.8058	Y	Y
3	Cs2RbSbBr6	1.2994	N	N	−0.8484	Y	Y
4	**Cs2NaBiBr6**	**0.7174**	**Y**	**N**	−0.8432	Y	Y
5	Cs2KBiBr6	0.9142	N	N	−0.8860	Y	Y
6	Cs2RbBiBr6	1.0474	N	N	−0.9317	Y	Y
7	Cs2NaSbCl6	1.3333	N	N	−1.0036	Y	Y
8	Cs2KSbCl6	1.3671	N	N	−1.0817	Y	Y
9	Cs2RbSbCl6	1.7458	N	N	−1.1066	Y	Y
10	Cs2NaBiCl6	1.1677	N	N	−1.0937	Y	Y
11	Cs2KBiCl6	1.3577	N	N	−1.1813	Y	Y
12	Cs2RbBiCl6	1.9255	N	N	−1.2019	Y	Y
13	Cs2NaSbI6	1.2048	N	N	−0.7630	Y	Y
14	Cs2KSbI6	0.9420	N	N	−0.8058	**Y**	**N**
15	Cs2RbSbI6	1.2464	N	N	−0.8484	**Y**	**N**
16	**Cs2NaBiI6**	**0.7065**	**Y**	**N**	−0.8432	Y	Y
17	Cs2KBiI6	0.8835	N	N	−0.8860	**Y**	**N**
18	Cs2RbBiI6	0.9172	N	N	−0.9317	**Y**	**N**
19	Cs2NaSbF6	1.3905	N	N	−1.1677	Y	Y
20	Cs2NaBiF6	1.3297	N	N	−1.2771	Y	Y
21	Cs2KBiF6	2.0333	N	N	−1.4035	Y	Y
22	Cs2KSbF6	1.9047	N	N	−1.2915	Y	Y
23	Cs2RbSbF6	2.1867	N	N	−1.3060	Y	Y
24	Cs2RbBiF6	2.5132	N	N	−1.4065	Y	Y

Notes: E_g_^a^ and ΔH_f_^a^ denote the classified criteria for solar cells from Ref. [10], E_g_^b^ and ∆ H^b^ denote the classified criteria for solar cells from Ref. [75]. Comparisons with DFT-calculated results are made to determine whether the classified results are solar cells (Y) or not (N), based on different criteria for stability and bandgap properties due to different calculation methods in the dataset. The different classified results were highlighted in bold.

**Table 10:** Classification performance comparison on the 918 double perovskite dataset.

Method	Accuracy (%)	F1 Score (%)	Precision
Bartel’s τ descriptor	91.00	—	—
Our method	96.22 (±1.01)	98.03 (±0.53)	96.89 (±0.83)
Improvement	+5.22%	—	—

Note: Values are reported as mean (± standard deviation) over 40 runs of 5-fold cross-validation. Bartel et al. [51] reported only accuracy for their τ descriptor.

## Data Availability

The original contributions presented in the study are included in the article, further inquiries can be directed to the corresponding authors.

## Supplementary Materials

The following supporting information can be downloaded at: https://www.mdpi.com/article/10.3390/ma19102018/s1, Table S1: The specific information of the base models of the stacking models and its corresponding performance on the test set with 40 runs of 5-fold cross-validation for E_g_ prediction. Table S2: The specific information of the base models of the stacking models and its corresponding performance on the test set with 40 runs of 5-fold cross-validation for H_f_ prediction.
