# Supplementary material for: Integrating NSGA-II and TOPSIS for Stacking Model Optimization in Pursuit of Halide Double Perovskite Screening
Source: Materials (Basel). 2026 May 12;19(10):2018. doi: 10.3390/ma19102018 (PMC13208370; doi:10.3390/ma19102018)
Supplement: Supplementary file 1 [file materials-19-02018-s001.zip › materials-4278660-supplementary.pdf]

# Supplementary

## Integrating NSGA-II and TOPSIS for Stacking Model Optimization in Pursuit of Halide Double Perovskite Screening

Guiqin Liang [1,2,*] and Jian Zhang[3,4,*]

1 College of Information and Communication, Guilin University of Electronic Technology, Guilin 541004, China

2 College of Physics and Electronic Information Engineering, Guilin University of Technology, Guilin 541004, China

3 College of Materials Science and Engineering, Guilin University of Electronic Technology, Guilin 541004, China

4 Guangxi Key Laboratory of Information Materials, Guangxi Collaborative Innovation Center of Structure and Property for New Energy and Materials, Guilin 541004, China

* Corresponding authors:

E-mail addresses: 2014054@glut.edu.com (G.L.), jianzhang@guet.edu.cn (J.Z.)

ORCID: 0000-0003-1289-2416 (G.L.)

**Table S1** The specific information of the base models of the stacking models and its corresponding performance on the test set with 40 runs of 5-fold cross-validation for $E_g$ prediction.

| No. | Num_Base_ Models | Base_Models | Meta_Model | MSE | RMSE | MAE | R2 |
|---|---|---|---|---|---|---|---|
| 1 | 3 | CatBR, Bag, LGBR | LR | 0.0362 | 0.1869 | 0.1110 | 0.9424 |
| 2 | 4 | Ada, BR, Bag, XGBR | LR | 0.0432 | 0.2048 | 0.1285 | 0.9309 |
| 3 | 5 | Ada, ETR, CatBR, Bag, RFR | LR | 0.0358 | 0.1861 | 0.1106 | 0.9429 |
| 4 | 6 | RFR, LGBR, Bag, XGBR, ETR, DTR | LR | 0.0435 | 0.2053 | 0.1297 | 0.9304 |
| 5 | 7 | BR, CatBR, DTR, XGBR, ETR, LR, Ada | LR | 0.0356 | 0.1853 | 0.1098 | 0.9433 |
| 6 | 8 | Ada, ETR, LR, BR, Bag, LGBR, DTR, RFR | LR | 0.0542 | 0.2297 | 0.1536 | 0.9131 |
| 7 | 9 | ETR, RFR, LR, Bag, XGBR, BR, Ada, DTR, LGBR | LR | 0.0433 | 0.2048 | 0.1291 | 0.9307 |
| 8 | 10 | ETR, Ada, RFR, LGBR, DTR, CatBR, XGBR, LR, BR, Bag | LR | 0.0361 | 0.1867 | 0.1106 | 0.9425 |
| 9 | 11 | GBR, ETR, Ada, RFR, LGBR, DTR, CatBR, XGBR, LR, BR, Bag | LR | 0.0359 | 0.1862 | 0.1101 | 0.9426 |

**Table S2** The specific information of the base models of the stacking models and its corresponding performance on the test set with 40 runs of 5-fold cross-validation for $H_f$ prediction.

| No. | Num_Base_ Models | Base_Models | Meta_Model | MSE | RMSE | MAE | R2 |
|---|---|---|---|---|---|---|---|
| 1 | 3 | CatBR, Bag, LGBR | LR | 0.00016 | 0.01178 | 0.00650 | 0.99716 |
| 2 | 4 | Ada, BR, Bag, XGBR | LR | 0.00017 | 0.01257 | 0.00765 | 0.99680 |
| 3 | 5 | Ada, ETR, CatBR, Bag, RFR | LR | 0.000136 | 0.010891 | 0.006088 | 0.997500 |
| 4 | 6 | RFR, LGBR, Bag, XGBR, ETR, DTR | LR | 0.00017 | 0.01224 | 0.00736 | 0.99693 |
| 5 | 7 | BR, CatBR, DTR, XGBR, ETR, LR, Ada | LR | 0.00015 | 0.01179 | 0.00615 | 0.99717 |
| 6 | 8 | Ada, ETR, LR, BR, Bag, LGBR, DTR, RFR | LR | 0.00017 | 0.01250 | 0.00754 | 0.99683 |
| 7 | 9 | ETR, RFR, LR, Bag, XGBR, BR, Ada, DTR, LGBR | LR | 0.00015 | 0.01166 | 0.00710 | 0.99718 |
| 8 | 10 | ETR, Ada, RFR, LGBR, DTR, CatBR, XGBR, LR, BR, Bag | LR | 0.00015 | 0.01147 | 0.00615 | 0.99729 |
| 9 | 11 | GBR, ETR, Ada, RFR, LGBR, DTR, CatBR, XGBR, LR, BR, Bag | LR | 0.00015 | 0.01166 | 0.00710 | 0.99718 |
